# Post genome-wide gene-environment interaction study: The effect of genetically driven insulin resistance on breast cancer risk using Mendelian randomization

**DOI:** 10.1371/journal.pone.0218917

**Published:** 2019-06-27

**Authors:** Su Yon Jung, Nicholas Mancuso, Jeanette Papp, Eric Sobel, Zuo-Feng Zhang

**Affiliations:** 1 Translational Sciences Section, Jonsson Comprehensive Cancer Center, School of Nursing, University of California, Los Angeles, Los Angeles, CA, United States of America; 2 Department of Pathology and Laboratory Medicine, David Geffen School of Medicine, University of California, Los Angeles, Los Angeles, CA, United States of America; 3 Department of Human Genetics, David Geffen School of Medicine, University of California, Los Angeles, Los Angeles, CA, United States of America; 4 Department of Epidemiology, Fielding School of Public Health, University of California, Los Angeles, Los Angeles, CA, United States of America; Duke Cancer Institute, UNITED STATES

## Abstract

**Purpose:**

The role of insulin resistance (IR) in developing postmenopausal breast cancer has not been thoroughly resolved and may be confounded by lifestyle factors such as obesity. We examined whether genetically determined IR is causally associated with breast cancer risk.

**Methods:**

We conducted Mendelian randomization (MR) analyses using individual-level data from our previous meta-analysis of a genome-wide association study (GWAS) (n = 11,109 non-Hispanic white postmenopausal women). Four single-nucleotide polymorphisms were associated with fasting glucose (FG), 2 with fasting insulin (FI), and 6 with homeostatic model assessment–IR (HOMA-IR) but were not associated with obesity. We used this GWAS to employ hazard ratios (HRs) for breast cancer risk by adjusting for potential confounding factors.

**Results:**

No direct association was observed between comprising 12 IR genetic instruments and breast cancer risk (HR = 0.93, 95% CI: 0.76–1.14). In phenotype-specific analysis, genetically elevated FG was associated with reduced risk for breast cancer (main contributor of this MR-effect estimate: *G6PC2* rs13431652; HR = 0.59, 95% CI: 0.35–0.99). Genetically driven FI and HOMA-IR were not significantly associated. Stratification analyses by body mass index, exercise, and dietary fat intake with combined phenotypes showed that genetically elevated IR was associated with greater breast cancer risk in overall obesity and inactive subgroups (single contributor: *MTRR/LOC729506* rs13188458; HR = 2.21, 95% CI: 1.03–4.75).

**Conclusions:**

We found complex evidence for causal association between IR and risk of breast cancer, which may support the potential value of intervention trials to lower IR and reduce breast cancer risk.

## Introduction

Postmenopausal women have an increased risk of developing breast cancer. Eighty percent of new breast cancer cases occur in women aged 50 and older, and obesity is a well-established risk factor for postmenopausal breast cancer [1–3]. The obesity–insulin resistance (IR) connections have been considered potential factors for cancer development. IR, leading to glucose intolerance, characterized by elevated fasting level of homeostatic model assessment–insulin resistance (HOMA-IR), hyperglycemia, and compensatory hyperinsulinemia, is thought to be crucial for the development of obesity-relevant cancers including postmenopausal breast cancer [4–6]. Mechanisms proposed for these associations include overexpression of insulin and insulin-like growth factor receptors [4,5,7] and dysregulation of multiple IR-signaling pathways [5,8–10], resulting in the enhanced anabolic state necessary for tumor growth and development. IR, thus may be associated with carcinogenesis.

The results of previous epidemiologic studies for the association between IR and postmenopausal breast cancer are inconsistent: rate ratio of HOMA-IR and glucose = 1.50 (95% confidence intervals [CIs]: 1.03–2.02 and 1.14–2.32, respectively) in a nested case-control study [11], hazard ratio (HR) of insulin = 2.40 (95% CI: 1.39–3.53 in a multicentric randomized controlled trial [12], and 95% CI: 1.30–4.41 in a case-cohort study [13]); and marginal [14] or no associations [15,16]. Those inconsistent findings may be partly due to potential selection bias, confounding effects by obesity and obesity-related lifestyle factors, short time exposures to biomarkers, measurement inconsistencies (e.g., different assays used to measure biomarkers), and reverse confounding or causation.

A Mendelian randomization (MR) analysis may be a better method to address these challenges. It has been used to analyze genetic variants as an instrumental variable to evaluate the effect of an exposure (e.g., IR) on an outcome (e.g., breast cancer risk) [17]. This genetic approach may help establish a relatively unbiased causal relationship between IR and breast cancer outcomes because MR reduces potential bias and confounding by random assortment of alleles at the time of gamete formation, resulting in a random assignment of exposure [17,18]. In addition, MR may eliminate short time exposure by incorporating a lifelong exposure to an allele (i.e., genetic variation randomly assigned at meiosis) [18]. MR can also prevent reverse causation because the random assignment of alleles precedes the phenotype and clinical outcomes [18,19].

In this study, we conducted MR analysis by using our previous genome-wide association study (GWAS) data to test the hypothesis that genetically determined IR has a potential causal effect on postmenopausal breast cancer risk.

## Materials and methods

### Data sources and selection of candidate instrumental variables

We used data from our previous meta-analysis of a genome-wide gene-environment (G*E) interaction study [20], which included 11,109 non-Hispanic white postmenopausal women enrolled in the Women’s Health Initiative (WHI) Harmonized and Imputed GWASs. Detailed rationale and design of the studies have been described elsewhere [21,22]. Briefly, the WHI study included postmenopausal women enrolled between 1993 and 1998 at 40 clinical centers across the US. Eligible women were 50–79 years old, postmenopausal, expected to live near the clinical centers for at least 3 years after enrollment, and able to provide written informed consent. The Harmonized and Imputed studies involved 6 GWASs. The genotyped data collected from the 6 GWASs were normalized to the reference panel GRCh37, and genotype imputation was performed using 1,000 genome reference panels [22]. Single-nucleotide polymorphisms (SNPs) with ${\hat{R}}^{2}\geq 0.6$ imputation quality were included in the G*E GWAS meta-analysis. The study was approved by the institutional review boards of each participating clinical center of the WHI and the University of California, Los Angeles.

Using the meta-analysis of G*E GWAS for IR and breast cancer risk, we identified IR-associated SNPs at genome-wide significance (p < 5 x 10^−8^) as instrumental variables and pruned the list of such SNPs by linkage disequilibrium (LD) (r^2^ < 0.1). For each SNP, we employed results obtained from this GWAS in multiple Cox regression analysis for breast cancer risk by adjusting for covariates. The covariates were selected for their association with IR and breast cancer from stepwise regression analyses: age, education, family income, family history of breast cancer, depressive symptoms, smoking, exercise, alcohol intake, percentage of calories from saturated fatty acids, body mass index (BMI), waist-to-hip ratio, hysterectomy, ages at menarche and menopause, use of oral contraceptives, exogenous estrogen only, and estrogen plus progestin.

Of the 58 SNPs associated with IR phenotypes in the women overall or the women stratified by obesity, physical activity, and high-fat diet, we finally identified 4 independent SNPs associated with fasting glucose (FG; 1 in overall, 2 in active, and 1 in high-fat diet groups); 2 independent SNPs associated with fasting insulin (FI; 1 in obese and 1 in inactive groups); and 6 independent SNPs associated with HOMA-IR (2 in overall, 2 in low-fat diet, and 2 in high-fat diet groups).

### Statistical analysis

Before conducting our MR analyses, we checked whether our data met basic assumptions required for valid inference. MR analysis typically assumes that genetic instruments are not weak (i.e., little explaining of the relevant phenotype). We estimated a sum of the T-squared statistics across phenotype-specific SNPs, assessing whether our SNP instruments were well powered for downstream MR analysis. With a threshold of 10, which is commonly used [23], we considered our SNPs as having sufficient strength (sum of the T-squared statistics: FG, 94.7; FI, 38.0; HOMA-IR, 126.1; and overall, 363.5). We also estimated the variance (%) of each trait explained by its associated variants (FG, 0.11%; FI, 0.28%; HOMA-IR, 5.05%; and overall, 5.45%).

MR studies may be confounded when modeled SNPs exhibit biological pleiotropy, or when SNPs independently affect breast cancer risk through intermediate traits other than IR. To determine the extent of pleiotropic signal in our study we conducted the following analyses. 1) Given obesity’s established role for breast cancer risk, we interrogated for the possible association of obesity [24] with the identified SNPs to exclude from the MR analysis; no SNPs showed evidence of pleotropic association with obesity; and 2) We performed a MR-Egger regression analysis [25] to test for directional pleiotropy, where the pleiotropic effect across SNPs on outcome is skewed in one direction rather than being balanced; no significant directional pleiotropy for any of the tested associations was observed.

Having demonstrated that our genetic instruments are predictive for respective phenotypes and unlikely to be confounded by pleiotropic effects, we performed MR analysis using the inverse-variance weighted method [26]. This quantifies the genetically determined association between IR and breast cancer risk. We took into consideration a correlation that could occur when exposure and outcome were assessed within the same population; thus, the MR estimates were adjusted for Spearman correlation between each IR phenotype and breast cancer risk. For the individual instrumental effects of IR on breast cancer risk, we calculated the ratio of β coefficients (= β_breastcancer_ / β_IR_) [18]. The results were reported as risk ratios and 95% CIs for the change in breast cancer risk per unit increase in log-odds of IR (i.e., the change in relative cancer risk [exponentiation of β] for women with IR compared with that for women without IR).

The heterogeneity of the MR estimate, which is additional evidence of pleiotropy, was evaluated by using Cochran’s Q test. A 2-tailed P value < 0.05 was considered statistically significant. A multiple-comparison adjustment was conducted by using the Benjamini-Hochberg method [27]. R3.5.1 was used.

## Results

The 12 IR SNPs identified for the different subgroups in our previous G*E GWAS are presented in Table 1. Of note is that *G6PC* rs13431652 of the FG SNPs and *PABPC1P2* rs77772624 and *LINC00460* rs17254590 of the HOMA-IR SNPs were observed at genome-wide significance in the overall and the high-fat diet groups.

**Table 1:** Characteristics of SNPs for the effect of IR on breast cancer risk.

Gene	SNP	Chr	Position	Allele	Alternative allele frequency		IR			Breast cancer risk	
				Ref/Alt	Controls (n = 10,520)	Breast cancer (n = 589)	OR	P	Q	HR (95% CI)	P
Fasting glucose											
G6PC2*	**rs13431652**	**2**	**169753415**	**T / C**	0.30	0.33	**0.79**	**6.99E-09**	**0.706**	**1.13 (1.00–1.28)**	**0.047**
***G6PC2***§	**rs13431652**	**2**	**169753415**	**T / C**	0.30	0.33	**0.77**	**1.08E-09**	**0.775**	1.13 (1.00–1.29)	0.059
***MKLN1***†	**rs117911989**	**7**	**130969793**	**G / A**	0.05	0.05	**1.98**	**3.97E-08**	**0.209**	0.74 (0.44–1.24)	0.250
***NKX2-2***†	**rs7273292**	**20**	**21473362**	**T / C**	0.01	0.0001	**3.37**	**4.35E-08**	**0.148**	0.66 (0.25–1.77)	0.407
Fasting insulin											
***NR5A2***¶	**rs10919774**	**1**	**199907716**	**G / A**	0.95	0.95	**1.98**	**2.53E-08**	**0.726**	1.34 (0.83–2.15)	0.226
***MTRR/LOC729506***€	**rs13188458**	**5**	**8127831**	**G / T**	0.75	0.77	**1.33**	**3.21E-08**	**0.435**	**1.25 (1.01–1.56)**	**0.043**
HOMA-IR											
PABPC1P2*	**rs77772624**	**2**	**147499474**	**A / C**	0.002	0.002	**29.65**	**4.96E-09**	**0.634**	0.61 (0.09–4.36)	0.623
***PABPC1P2***§	**rs77772624**	**2**	**147499474**	**A / C**	0.002	0.002	**28.92**	**9.36E-09**	**0.711**	0.61 (0.09–4.34)	0.620
***MSC***¥	**rs13277245**	**8**	**72606942**	**A / G**	0.18	0.17	**29.57**	**4.92E-08**	N/A	0.87 (0.48–1.60)	0.661
***DOCK1***¥	**rs113847670**	**10**	**128874679**	**C / T**	0.03	0.04	**9.18**	**2.85E-08**	**0.571**	0.49 (0.12–2.00)	0.320
LINC00460*	**rs17254590**	**13**	**107037344**	**G / C**	0.02	0.0004	**2.52**	**2.40E-08**	**0.620**	1.00 (0.55–1.83)	0.999
***LINC00460***§	**rs17254590**	**13**	**107037344**	**G / C**	0.02	0.0004	**2.67**	**8.86E-09**	**0.882**	1.09 (0.60–1.98)	0.784

Alt, alternative allele; Chr, chromosome; CI, confidence interval; HOMA-IR, homeostatic model assessment–insulin resistance; HR, hazard ratio; IR, insulin resistance; N/A, not available; OR, odds ratio; Q, Cochran’s Q; Ref, reference allele; SNP, single–nucleotide polymorphism. Numbers in bold face are statistically significant.

* SNPs at genome-wide level identified in overall analysis.

§ SNPs at genome-wide level in subgroup analysis: identified in high-fat diet group (calories from saturated fatty acids [SFA] ≥ 7.0%).

† SNPs at genome-wide level in subgroup analysis: identified in active group (metabolic equivalent [MET] ≥ 10).

¶ SNPs at genome-wide level in subgroup analysis: identified in obese group (body mass index ≥ 30.0 kg/m^2^).

€ SNPs at genome-wide level in subgroup analysis: identified in inactive group (MET < 10).

¥ SNPs at genome-wide level in subgroup analysis: identified in low-fat diet group (calories from SFA < 7.0%).

**Fig 1:**
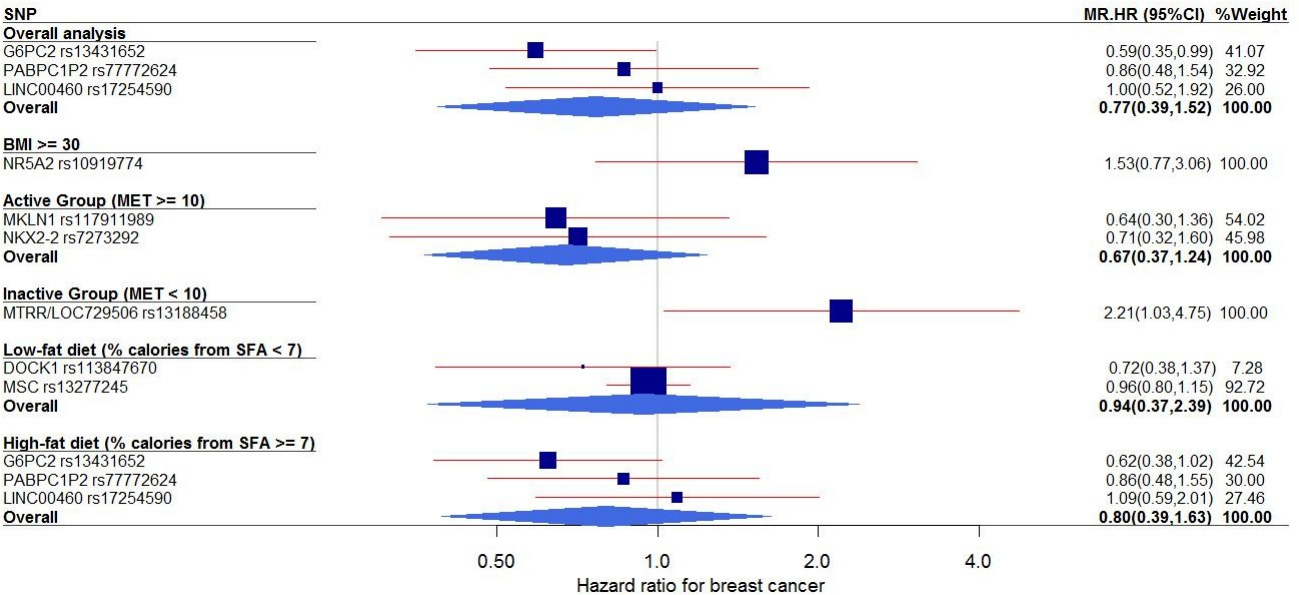
Forest plot of the MR effects of IR on breast cancer risk in overall group and subgroups. For each of non-pleiotropic IR SNPs, the plot shows the effects of genetically elevated IR (FG, FI, or HOMA-IR) on breast cancer risk in the overall group and subgroups, presented as the 95% CIs (indicated with red lines) of the estimates and the inverse-variance weights (percentages proportional to the size of the blue squares). BMI, body mass index; CI, confidence interval; FG, fasting glucose; FI, fasting insulin; HOMA-IR, homeostatic model assessment–insulin resistance; HR, hazard ratio; IR, insulin resistance; MET, metabolic equivalent; MR, Mendelian randomization; SFA, saturated fatty acids; SNP, single–nucleotide polymorphism.

An MR analysis for the association between individual genetic instruments for each phenotype (FG, FI, and HOMA-IR) and breast cancer risk (adjusted by covariates) identified 2 SNPs whose genetically driven IR phenotype was associated with breast cancer outcome (Table 2 and Fig 1): in the overall analysis, *G6PC* rs13431652 (HR = 0.59, 95% CI: 0.35–0.99); and in the inactive subgroup, *MTRR/LOC729506* rs13188458 (HR = 2.21, 95% CI: 1.03–4.75). After a multiple-testing correction, *MTRR/LOC729506* rs13188458 remained statistically significant (p value after a multiple-comparison adjustment = 0.043). In the MR analysis of the combined effects of genetic instruments on breast cancer risk by phenotype (Table 2), the pooled estimate of genetically predicted FG was associated with decreased breast cancer risk (HR = 0.63, 95% CI: 0.50–0.79, p value after a multiple-comparison adjustment = 0.036), whereas those of genetically driven FI and HOMA-IR were not significantly associated.

**Table 2:** Mendelian randomization analysis of the effect of IR on breast cancer risk.

Subgroup	Fasting glucose			SNP	Fasting insulin			SNP	HOMA-IR			SNP
	HR (95% CI) ¶*	P	P_hat_	n	HR (95% CI) ¶*	P	P_hat_	n	HR (95% CI) ¶	P	P_hat_	n
**Overall**	**0.59 (0.35–0.99)**	**0.047**	N/A	1					0.92 (0.37–2.30)	0.460	0.747	2
**BMI ≥ 30**					1.53 (0.77–3.06)	0.226	N/A	1				
**Active group (MET ≥ 10)**	0.67 (0.37–1.24)	0.077	0.865	2								
**Inactive group (MET < 10)**					**2.21 (1.03–4.75)**	**0.043**	N/A	1				
**Calories from SFA < 7.0%**									0.94 (0.35–2.50)	0.565	0.406	2
**Calories from SFA ≥ 7.0%**	0.62 (0.38–1.02)	0.059	N/A	1					0.96 (0.22–4.23)	0.807	0.591	2
**Pooled estimate**	**0.63 (0.50–0.79)**	**0.012**	0.931	4	1.80 (0.18–18.06)	0.190	0.494	2	0.94 (0.81–1.08)	0.236	0.851	6

BMI, body mass index; CI, confidence interval; HOMA-IR, homeostatic model assessment–insulin resistance; HR, hazard ratio; IR, insulin resistance; MET, metabolic equivalent; SFA, saturated fatty acids; SNP, single–nucleotide polymorphism. Numbers in bold face are statistically significant. Note: P_hat_ was estimated on the basis of Cochran’s Q.

¶ The Mendelian randomization HR has been estimated by adjusting for Spearman correlation between each phenotype and breast cancer risk within the same population.

* The Mendelian randomization effect of single SNPs on breast cancer risk has been estimated via the ratio of β coefficients (= β_breastcancer_ / β_IR_) (18).

**Fig 2:**
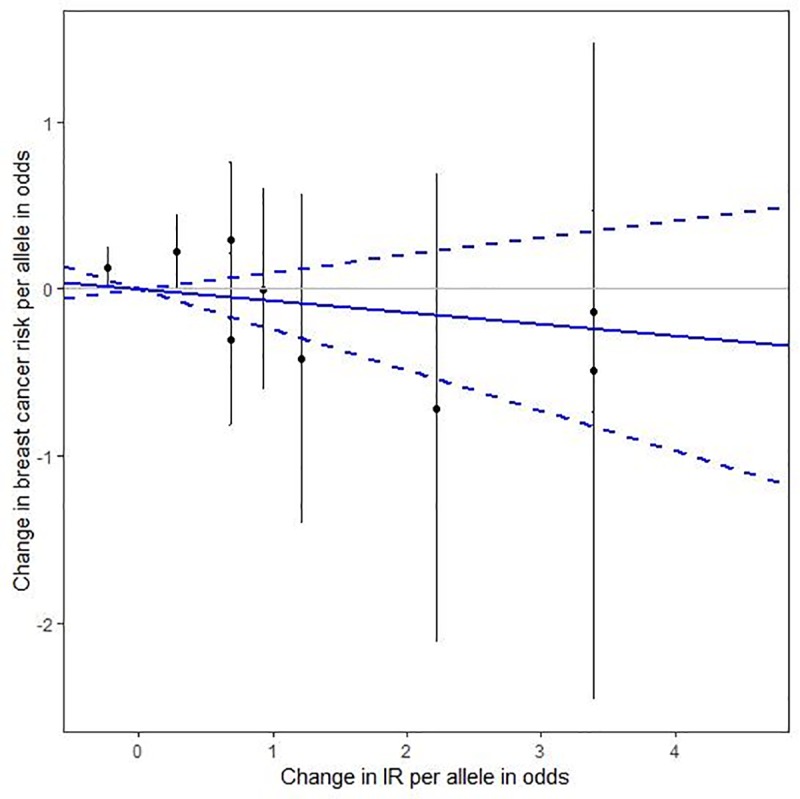
The effect of individual genetic instrumental variables for IR on breast cancer risk. Each black dot reflects a genome-wide IR-elevating genetic variant. The blue lines indicate regression and 95% CIs of IR on breast cancer risk (HR = 0.93, 95% CI: 0.76–1.14). CI, confidence interval; HR, hazard ratio; IR, insulin resistance.

We also performed subgroup analyses (Fig 1) stratified by BMI, exercise, and dietary fat intake. In the overall obesity (BMI ≥ 30) and inactive (metabolic equivalent [MET] < 10) subgroups, genetically elevated IR was associated with increased risk for breast cancer, while in the active subgroup (MET ≥ 10), genetically raised IR was associated with reduced risk for breast cancer, although the relationship in this active subgroup was not statistically significant. By combining all the IR-related SNPs, we performed an overall pooled MR analysis (Fig 2) and observed no evidence of a genetically predicted association between IR and breast cancer risk (HR = 0.93, 95% CI: 0.76–1.14).

We performed an MR-Egger test to detect potential directional pleiotropy and found no significant evidence of apparent directional pleiotropy across the tested associations. We further conducted a sensitivity test for the association between genetic instrumental variables for IR and breast cancer risk by replacing current HRs for breast cancer with HRs that were adjusted for age and 10 genetic principal components only; similar results were observed and no evidence of apparent directional pleiotropy was observed.

## Discussion

We conducted genetic analyses for IR phenotypes in relation to postmenopausal breast cancer risk in an MR framework, which could establish potential causality. If genetic instruments are not linked to the outcomes through any alternative pathway, the results of an MR study could resemble those of randomized clinical trials [17] and provide a robust causal inference. The key point of our study is the inclusion of nonoverlapping IR and obesity/obesity-lifestyle genetic variants, suggesting the pleiotropic exclusion. MR study also reflects lifelong exposures, providing the long-standing effect of IR on breast cancer risk, and it is less subject to reverse causality than an observational study. Although our MR study was not designed to elaborate biological mechanisms, our findings indicate that lifetime exposure to IR is likely to influence the development of breast cancer in postmenopausal women.

Particularly, in the phenotype-specific analysis, genetically elevated FG was associated with a reduced risk for breast cancer. Previous prospective [14] and MR studies [17] showed no association between FG and risk of breast cancer, explaining that FG reflects glycogenolysis activity in hepatic insulin sensitivity and represents a relatively short-term phenomenon of IR. In contrast, 2-hour glucose levels are associated with a greater risk of breast cancer, reflecting beta cell function and skeletal muscle insulin sensitivity, thus representing relatively long-term exposure to IR. In our MR analysis, *G6PC2* rs13431652 was the main contributor of the MR effect estimate of FG on breast cancer. *G6PC2* encodes the glucose-6-phosphatase catalytic 2 subunit. It regulates glycemia by opposing the action of glucokinase in pancreatic beta cells, thus modulating glycolytic flux and glucose-stimulated insulin secretion [28]. Individuals with this genetic mutation (related to type 2 diabetes [T2DM]) have mild hyperglycemia from birth onwards, and the early diagnosis of the pre-diabetic condition leads to the treatment of other potential cancer risk factors, such as hypercholesterolemia, thus conferring additional protection against breast cancer later in life [29].

In addition, our MR analysis of individual genetic instruments of FI indicated that rs13188458 in an intergenic region of *MTRR/LOC729506* was a strong contributor to the effect of genetically driven FI on increased risk of breast cancer; this association was observed only in the subgroup of inactive women. Mutations in *MTRR* can induce IR and T2DM in adipose tissue by provoking endoplasmic reticular stress, resulting in inhibited insulin signaling [30]. Previous studies reported the association of this genetic mutation with lung and colorectal cancers [31,32], but not with breast cancer; this suggests that the incorporation of obesity-related lifestyle factors (e.g., physical activity) in the analysis is critical.

Our study results should be interpreted with caution because of unmeasured confounding factors that could have introduced bias. MR analysis requires several assumptions, such as LD (i.e., SNP instruments may not be correlated with another SNP), weak genetic instruments, pleiotropy, and population structure (i.e., results biased due to tagged environmental factors) [33]. In the current analysis, we properly addressed LD and weak genetic instruments by pruning correlated genetic variants and including only those with strong association signal for phenotype. Next we reduced pleiotropic effect driven by obesity in two ways. First, in our previous GWA G*E analysis for IR and breast cancer risk, we conducted stratification analyses by obesity and related lifestyle factors, so the effects of such modifiers were removed before we performed this MR analysis. Second, by using HRs for breast cancer that were adjusted by lifestyle and reproductive factors, our MR analysis examined the effect of genetically driven IR on breast cancer risk adjusted by those potential confounding factors. Nevertheless, residual confounding may have affected our study results. Further, if obesity acts upstream of IR, so the effect that mediates between IR and breast cancer via obesity is substantial, excluding obesity could make the MR estimates (i.e., the direct effect of IR on breast cancer risk) less reliable. Finally, we reduced the potential for population structure bias since we adjusted for the correlation between IR and breast cancer risk using individual-level exposure and outcome data from the same study population.

MR analysis might also be subject to nonlinearity between exposure and outcome. The association between genetically driven IR and cancer risk may be influenced by the feedback mechanism (canalization), resulting in nonlinear processes, but such canalization tends to bias MR estimates toward the null, so it is unlikely to alter the statistical directions or significance [34]. Our study could have overfit the analysis because the data on exposure and outcome were gathered from the same population. Finally, our findings should not be extrapolated to other populations because our study population was limited to non–Hispanic white postmenopausal women.

In conclusion, we quantified the potential causal relationship between genetically elevated IR and risk of breast cancer and found complex evidence that lifetime exposure to IR is likely to influence the development of breast cancer in postmenopausal women. Further biologic research into this complicated association of IR with breast cancer by incorporating different behavior types may help clarify the mechanisms underlying the associations observed in our study. Nonetheless, our findings may provide additional evidence for conducting intervention trials to lower IR, thus reducing breast cancer risk.
